# A New Model for Open Sharing: Massachusetts Institute of Technology's OpenCourseWare Initiative Makes a Difference

**DOI:** 10.1371/journal.pbio.0020200

**Published:** 2004-08-17

**Authors:** Anne H Margulies

## Abstract

M.I.T. offers an education to the world through its OpenCourseWare program

Imagine a fledgling biology instructor at a university in the developing world. Heading into her first semester of teaching, she is armed with nothing but her college degree, some old notebooks, and—if she is lucky—a late-edition textbook. Forging a curriculum that is both current and engaging for her students could be a daunting challenge.

But what if that same young instructor was given free and open access to a syllabus, complete lecture notes, and problem sets and solutions from two members of the faculty of the Massachusetts Institute of Technology (MIT)? And not just any faculty members, but David Page—the recipient of a MacArthur Foundation Prize Fellowship in 1986, a Searle Scholar's Award in 1989, and the Amory Prize for advances in reproductive biology from the American Academy of Arts and Sciences in 1997—and Chris Kaiser, who won a 1999 fellowship from MIT that recognizes his teaching excellence?

This is the premise of MIT's OpenCourseWare project.

Utilizing the Internet, MIT OpenCourseWare (MIT OCW) has opened MIT's curriculum and educational materials to a global audience of teachers and learners—an instructor at a new engineering university in Ghana, a precocious highschool biology student in suburban Chicago, a political scientist in Poland, a literature professor in upstate New York—who are all now able to use the same materials that MIT's professors rely on to teach their full-time students.

## The Makings of a Movement

Ten years from now, we expect that MIT OCW will have become firmly planted in MIT's educational landscape. But MIT OCW was just a leap of faith when the concept was originally proposed by a group of faculty four years ago. In the fall of 1999, Provost Robert A. Brown asked the faculty committee to provide strategic guidance on how the institute should position itself in the e-learning environment. At first, many members of the group assumed that their work would lead to an “MIT.com” venture. But after a year of analysis, market research, and development of business scenarios, the committee concluded that a revenue-generating distance-education model was not desirable for MIT.

The committee went back to the drawing board and, convinced that open software and open systems were the wave of the future, came to a very simple conclusion: that MIT should use the Internet to give its teaching materials away.

Brown and MIT President Charles Vest instantly recognized the simplicity and brilliance of the idea. “It seemed to me that it would be a way to advance education, by constantly widening access to our information and inspiring other institutions to do the same with theirs,” Vest said.

While the 701 courses currently available represent just a third of the ultimate goal of 2,000 courses by the year 2008, MIT OCW has already had an impact on MIT's campus. We have published teaching materials from almost half of MIT's 950 faculty members, and a significant portion of the faculty have told us that they are already using materials available on MIT OCW—the lecture notes, syllabi, problem sets, and exams of their colleagues—to prepare for their classes, do research, and help their students.

But the real payoff of what we hope will become the “opencourseware movement” will be its effect on educators and learners around the world. Our goal is to create a model that other universities can follow and improve upon. Ultimately, the trend toward open knowledge will help bring people of all backgrounds together and promote improved educational systems across the globe.

## Measuring Success

Since April 2001, we have received more than 20,000 e-mail messages from around the world endorsing the vision and potential benefits of sharing knowledge freely. A typical message came from Andrew Wilson in the United Kingdom in October 2003: There can be no greater hope for humankind than the belief that wisdom generated through increased learning will ultimately lead to a better world. With OCW, MIT has taken an ethical stand against the belief that knowledge should only be accessible to those who can pay for it or are in proximity to it.”

Just after its “official launch” in fall 2003, MIT began a rigorous data collection process to find out who is accessing MIT OCW, why and how they use it, and what difference the initiative makes. The results of this first baseline evaluation confirm what we have heard anecdotally through those e-mails: that educators, students, and self-learners around the world are using our course materials, and that, overwhelmingly, they find the materials useful in meeting their own learning and teaching goals.

### Who Is Accessing MIT OCW?

On average, MIT OCW clocks over 11,000 visits per day, with nearly a quarter-million unique visitors per month. About 45% of these visitors are from the United States and Canada. Outside North America, the top countries of origin are China, the United Kingdom, Germany, India, and Brazil. About 52% of visitors identify themselves as “self-learners,” 31% as “students” enrolled in a formal course of study, and 13% as “educators.” We view educators as a particularly important target audience because it is through them that MIT course materials can touch the greatest number of people and have the most profound impact on education around the world.

### Why and how are they using it?

MIT OCW asked visitors their primary purpose in using MIT course materials. Of educators who responded, about 57% answered that they use it for course or curriculum development, 33% to enhance their subject matter understanding or support research, and 7% for student advising. Elements of MIT materials have been adapted for classroom use by 47% of educators who answered our survey, while 41% report they are considering it.

## Critical Mass

**Figure 1 pbio-0020200-g001:**
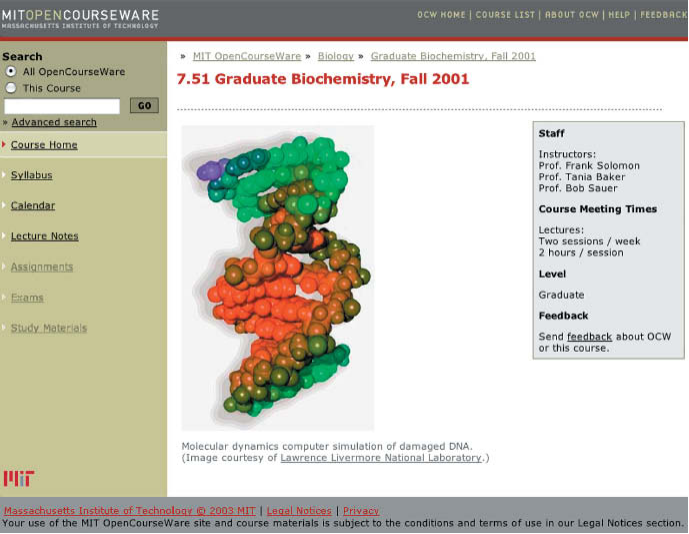
Sample MIT OCW Course Homepage for Graduate Biochemistry Course (Image of DNA courtesy of Lawrence Livermore National Laboratory.)

Among the 33 academic disciplines available are 15 courses from the MIT Department of Biology (see Figure 1), 63 from the Department of Brain and Cognitive Sciences, and 13 from the Harvard–MIT Division of Health Sciences and Technology.[Fig pbio-0020200-g001]

Educators, students, and self-learners from a wide variety of fields will find materials they can use in their teaching and learning activities. And as the opencourseware concept spreads to other colleges and universities, we expect that access to the work of faculty from diverse disciplines and institutions will increase, by an order of magnitude, the benefits to educators and learners who (whether for reasons of geography, cost, or culture) would not otherwise have access to such materials.

History has proved that education and discovery are best advanced when knowledge is shared openly. Our agenda must evolve to shape the future, and to respond to new challenges and opportunities.

At MIT, we believe the idea of opencourseware is one such opportunity, which we must seize during the next decade.

*For more information about MIT OCW, please contact Jon Paul Potts, MIT OCW Communications Manager, at E-mail:* jpotts@mit.edu or 617-452-3621.

## Abbreviations

MIT: Massachusetts Institute of Technology
MIT OCW: MIT OpenCourseWare

